# Frequency of Egg Intake Associated with Mortality in Chinese Adults: An 8-Year Nationwide Cohort Study

**DOI:** 10.3390/ijerph192214777

**Published:** 2022-11-10

**Authors:** Ke Wang, Qianqian Xiang, Lan Hu, Lu Wang, Yunquan Zhang

**Affiliations:** 1 Department of Nursing, Medical College, Wuhan University of Science and Technology, Wuhan 430065, China; 2 Hubei Provincial Center for Disease Control and Prevention, Wuhan 430079, China; 3 Department of Head and Neck Surgery, Hubei Cancer Hospital, Tongji Medical College, Huazhong University of Science and Technology, Wuhan 430079, China; 4 School of Basic Medicine, Hubei University of Arts and Sciences, Xiangyang 441053, China; 5 Department of Epidemiology and Biostatistics, School of Public Health, Wuhan University of Science and Technology, Wuhan 430065, China; 6 Hubei Province Key Laboratory of Occupational Hazard Identification and Control, Wuhan University of Science and Technology, Wuhan 430065, China

**Keywords:** egg consumption, all-cause mortality, cohort, Chinese

## Abstract

Whether egg consumption plays a beneficial/detrimental role in affecting human health and longevity has been debated for decades. Large-scale cohort evidence from low- and middle-income populations are scarce. In this study, we aimed to assess the association of egg consumption with mortality in Chinese adults. A nationwide cohort of 30,835 participants ages 16–110 years were enrolled from 25 provincial regions in China’s mainland. Dietary intake (e.g., egg, meat, vegetable) was assessed by a food-frequency questionnaire. Cox proportional hazards models were used to examine associations between egg consumption and mortality, adjusting for demographic characteristics, dietary factors and health status. Dose–response relationships were investigated using the smoothing function of restricted cubic splines. Several subgroup analyses were performed. A total of 1651 all-cause deaths occurred during a median follow-up of 8.1 years. Egg consumption was associated with lower risks of mortality, with the lowest risk occurring in the group of moderate egg intake (3–6 times/week). Compared with non-consumers, the hazard ratios (95% confidence intervals) for mortality were 0.84 (0.72–0.97) for 3–6 times/week and 0.82 (0.69–0.98) for ≥7 times/week, whereas no significant associations were observed among the lower egg intake group (1–2/week). An approximately inverted J-shaped association was observed in three models, while restricting our analysis in the multivariable model (model 3) did not identify a significant violation for the linear relationship (*p* for nonlinear = 0.122). There were no statistically significant effect modifications in the subgroup analyses. Egg consumption may be associated with lower risks of mortality in Chinese adults. Our findings found moderate-to-high egg consumption might be beneficial for improving long-term health and longevity.

## 1. Introduction

Dietary risk factors have raised significant attention worldwide over the past decades [1]. According to the Global Burden of Disease Study in 2019, 7.9 million deaths and 187.7 million disability-adjusted life-years (DALYs) could be attributable to dietary risk factors worldwide [2]. As a common and rich in nutrients food item, the egg contains high-quality protein, several vitamins and micronutrients, which could contribute to the overall health of the public [3]. Meanwhile, the high cholesterol content in eggs motivates more caution among the general population, and some epidemiologic studies have associated higher egg consumption (especially cholesterol in yolk) with a wide range of morbidity and mortality outcomes [4,5,6]. Due to the dual role of egg intake on public health, there is no consensus on egg intake recommendations across countries.

Inconsistent associations between egg intake and mortality were reported in accumulating longitudinal studies, wherein existing research was predominantly conducted in developed regions such as the U.S. [7,8] and European countries [9,10,11]. A pooled analysis of three large prospective cohort studies involving 202,429 participants observed that an intake of ≥7 eggs/week was associated with a 7% increased risk in all-cause mortality among Black Americans [12]. In Italy, a longitudinal analysis on 20,562 adults aged ≥ 35 years reported an increased risk of all-cause mortality associated with intake of more than 4 eggs/week [10]. As one of an ongoing multi-centric cohort study conducted in ten European countries, the Spanish study found a 41% reduction between egg intake and deaths from the nervous system, while no significant association was observed with all-cause and specific-cause mortality [9]. To date, even a recent systematic review and dose–response meta-analysis of egg-mortality associations did not provide conclusive scientific evidence on the role of egg consumption on human health outcomes [13]. Due to great differences in dietary habits between Chinese and western populations and the lack of nationally representative cohort evidence in China, great research gaps and uncertainties still exist in the relationship between egg intake and mortality among Chinese adults.

Here, we conceived a nationally representative cohort study covering 25 provincial regions to examine the relationship between dietary intake of eggs and all-cause mortality in China’s mainland. We also sought to determine whether the association was linear or nonlinear over the whole exposure range and whether it varied across subgroups of the population.

## 2. Methods

### 2.1. Study Design and Participants

**Figure 1:**
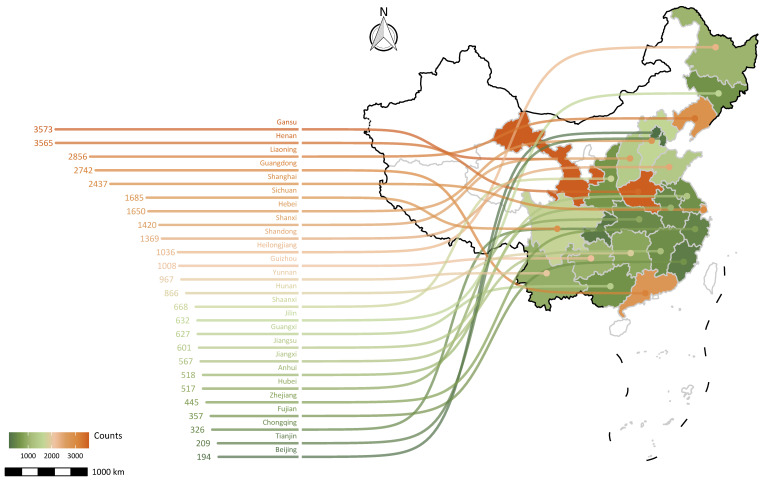
The map of provincial distribution of adult samples (*n* = 30,835) at baseline.

All data were obtained from the China Family Panel Study (CFPS), a nearly nationwide and stratified multistage probability sampling survey to track sociodemographic and economic patterns and health status in China. The sample covered a broad range of China’s mainland, including 25 provinces or municipalities and autonomous regions (Figure 1). The CFPS is a prospective longitudinal survey involving >30,000 adult men and women aged 16 to 110 years and enrolled between April 2010 and February 2011. Since baseline (CFPS 2010), the CFPS has released four complete waves of follow-up surveys (CFPS 2012, 2014, 2016, and 2018). The standard questionnaire information was obtained and updated by trained investigators conducting face-to-face interviews with the help of computer-assisted personal interviewing (CAPI) technology. The Peking University Biomedical Ethics Review Committee approved the underlying protocol (IRB00001052-14010), and all study participants provided written informed consent before enrollment. The data are publicly available and can be accessed at the CFPS official website (https://opendata.pku.edu.cn/dataverse/CFPS/en, accessed on 13 August 2021). Detailed descriptions of study design and methods are reported elsewhere [14,15].

The current analyses included the baseline (CFPS 2010) survey and the following four survey waves (CFPS 2012–2018) of the CFPS adult datasets, with a total of 33,600 participants who completed questionnaires at baseline. We used the following exclusion criteria to examine the longitudinal association between egg consumption and all-cause mortality: (1) loss to follow up since the first wave (CFPS 2010) of the follow-up survey (*n* = 2549), (2) lack of death information at consequent follow-up surveys (*n* = 191), and (3) incompleteness of important covariates (*n* = 25). Finally, a total of 30,835 participants (14,970 men and 15,865 women) were included for the cohort analysis (Figure S1).

### 2.2. Assessment of Egg Consumption

Estimates of dietary intake information at baseline were derived from a self-reported food frequency questionnaire (FFQ) developed by the Institute of Social Science Survey (ISSS) of Peking University for CFPS (http://www.isss.pku.edu.cn/cfps/wdzx/tcwj/index.htm, accessed on 13 August 2021). The FFQ inquired about the consumption frequency of each food item per week on average in the last three months by including: (1) unprocessed red meat items (pork, beef, lamb, etc.) and processed (bacon, ham, sausage, etc.), (2) all types of vegetables (plants and fungus), (3) fish intake (a variety of aquatic products), (4) dairy products (cow milk, goat milk and its processed products). According to the answers, egg consumption frequency was divided into the following four categories: 0, 1–2, 3–6 and ≥7 times eating egg per week. The Cronbach’s alpha coefficient of the food frequency questionnaire was 0.822. The Kaiser–Meyer–Olkin (KMO) value was 0.800 with significant Bartlett’s Test of Sphericity (*p* < 0.001), indicating that the food frequency questionnaire had good reliability and validity.

### 2.3. Death Ascertainment

In this study, the primary outcome of interest was all-cause mortality. Vital status and cause of death of all CFPS participants was determined by the responses of family members to the interviewers during the follow-up survey (CFPS-2012, -14, -16, and 2018). Person-years of follow-up was calculated as a time interval from the date of the baseline questionnaire to death, loss to follow-up, or the end of this study (CFPS 2018).

### 2.4. Covariate Assessments

Baseline information on covariates was collected through the CFPS panel’s standard questionnaire for the current study. Covariates included sociodemographic characteristics (age, gender, ethnicity, body mass index (BMI), household income, urban or rural resident), lifestyles (physical activity, alcohol consumption, smoking), dietary factors (meat, vegetable, fish and dairy products), health status (history of chronic diseases). Previous studies from the CFPS database indicated that physical moderate exercise was defined as at least 1–60 min/day of moderate-intensity physical activity (MPA) [16]. Based on this study, physical activity in our study was classified as 0 min/week, 1–60 min/week and ≥60 min/week. BMI was calculated from the body weight in kilograms divided by height in meters squared (kg/m^2^) based on height and weight obtained from a standard questionnaire. Dietary intake was recorded in the same way as egg consumption and grouped according to the calculation of average intake for all participants in the CFPS 2010 codebook (https://www.isss.pku.edu.cn/cfps/sjzx/gksj/index.htm, accessed on 13 August 2021), frequency of meat intake was divided into three groups (0 times/week, 1–3 times/week, ≥4 times/week), vegetable intake (0 times/week, 1–7 times/week, ≥8 times/week), fish intake (0 times/week, 1 times/week, ≥2 times/week) and dairy products intake (0 times/week, 1–3 times/week, ≥4 times/week). Chronic diseases (e.g., circulatory, respiratory and digestive) were recorded according to the Disease Classification Codebook designed by Peking University [15].

### 2.5. Statistical Analysis

Baseline characteristics of all participants were presented as means (standard deviation, SD) or count (percentages, %) by predefined categories of egg intake frequency (0/week, 1–2/week, 3–6/week, ≥7/week). We estimated the hazard ratios (HRs) and 95% confidence intervals (CIs) for mortality associated with egg consumption using the lowest category of egg intake as the reference group (0/week). To account for potential spatial variation between provinces, we incorporated province-level random intercepts into Cox proportional hazard models. *P*-values of a possible linear trend (*p* for trend) were calculated by assigning each category of egg intake as a continuous variable. We further verified the proportional hazards assumption by estimating the weighted Schoenfeld residuals; after the proportional assumption was corrected by conducting age-, gender-, BMI-, ethnicity-, and smoking-stratified analyses, we detected no evidence of violation of the proportionality assumption (*p* value > 0.05) [7].

We established stepwise models to assess the association between egg intake and mortality: Model 1 was unadjusted; Model 2 was adjusted for age (16–44, 45–59 and ≥60 y) and gender (men or women); Model 3 was further adjusted for BMI (<18.5, 18.5–23.9 and 24+ kg/m^2^), ethnicity (Han or minority), residential region (urban or rural areas), household income (<15,000, 15,000–39,999 and 40,000+ ¥), physical activity (0 min/week, 1–60 min/week and ≥60 min/week), alcohol drinking (current, former and never), smoking status (current, former and never), chronic diseases (circulatory, respiratory, and digestive), frequency of meat intake (0/week, 1–3/week, ≥4/week), vegetables intake (0/week, 1–7/week, ≥8/week), fish intake (0/week, 1/week, ≥2/week) and dairy product intake (0/week, 1–3/week, ≥4/week). In our subgroup analysis, lifestyles such as physical activity were divided into two groups: yes (>1 min/week) or no (0 min/week); alcohol drinking (current and former) or no drinking (never); cigarette smoking (current and former) or no smoking (never).

We conducted a restricted-cubic-spline analysis to evaluate the associations of egg intake on all-cause mortality based on three models. To identify whether the observed associations were independent of potential effect modifiers, subgroup analyses were performed for age, gender, BMI, residential region, smoking, drinking and physical activity. The interaction terms (*p* for interaction <0.05) were tested to investigate the potential effect modifications via the likelihood ratio test [17]. A series of sensitivity analyses were performed to check the robustness of the results by excluding: (1) deaths within the initial first year of follow-up to decrease reverse causality bias, (2) cases aged less than 30 years, as the outcome of interest tends to occur at older ages [9], (3) participants with chronic diseases at baseline (such as circulatory, respiratory, digestive disease) to minimize the potential impact of pre-existing disease on exposure variables.

Statistical tests were two-sided, and *p* values < 0.05 were considered to determine statistical significance. All of the statistical analyses and visualization were completed using R version 4.0.2 (R Foundation for Statistical Computing, Vienna, Austria), using the “survival” package for stratified Cox modeling and the “rms” package for restricted-cubic-spline smoothing.

## 3. Results

Table 1 presents the summary characteristics of included participants by categories of egg consumption frequency. The mean (SD) age of the 30,835 participants was 45.6 (16.3) years, 48.5% were men and 91.7% were Han ethnicity. BMI was averaged at 21.5 (5.2) kg/m^2^, and overweight and obese participants (BMI ≥ 24 kg/m^2^) accounted for 27.1%. Within a median follow-up of 8.1 years (2,682,509 person-years), a total of 1651 all-cause deaths were ascertained. Among all the participants, 7269 (23.6%) were non-consumers, and 8561 (27.8%), 9445 (30.6%), 5560 (18.0%) subjects consumed 1–2, 3–6, and ≥7 times/week, respectively. In the higher intake group, more than a third (36.2%) of the participants had regular meat intake and about half (48.6%) had habitual intake of vegetables, whereas the consumption of fish and dairy products only accounted for 26.8% and 13.8% respectively.

**Figure 2:**
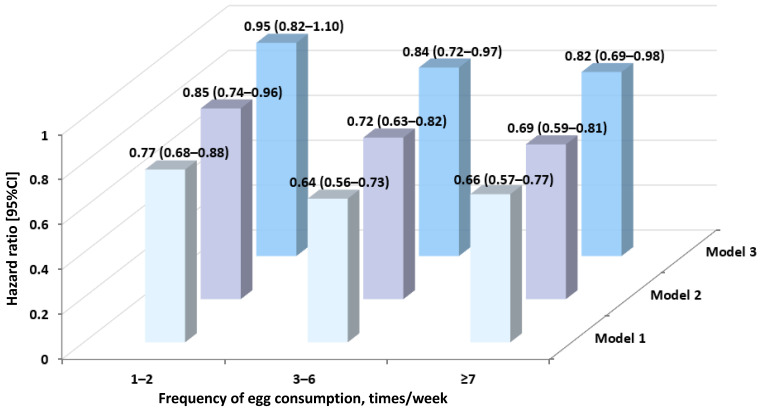
Association between egg consumption and all-cause mortality. Model 1: Unadjusted. Model 2: Model 1 + age + gender. Model 3: Model 2 + BMI, ethnicity, residential region, annual household income, physical activity, smoking status, alcohol drinking, chronic disease, meat intake, vegetable intake, fish, dairy products.

Figure 2 summarizes the associations of egg consumption with all-cause mortality. Highly comparable HR estimates were observed in three models. Compared with non-consumers, moderate-to-high egg intake frequency was found to be associated with a lower risk of mortality, while no significant associations were detected in the lower egg intake group (1–2 times/week). In the multivariate-adjusted model (model 3), HR estimates were 0.95 (0.82 to 1.10) for 1–2 times/week, 0.84 (0.72–0.97) for 3–6 times/week and 0.82 (0.69–0.98) for ≥7 times/week, respectively, exhibiting a significant trend (*p* for trend < 0.01) between frequency of egg intake and the risk of mortality (Table S1).

**Figure 3:**
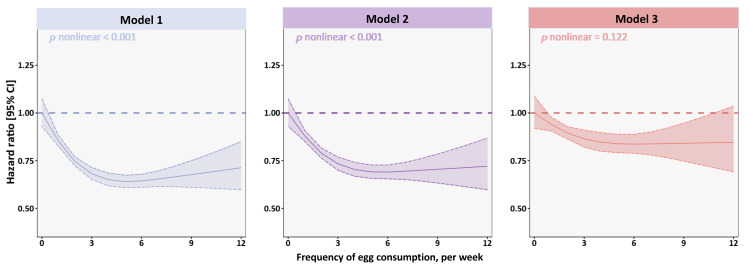
Restricted-cubic-spline curves detailing egg intake frequency associated with the risk of all-cause mortality. Solid lines represent point estimates of the hazard ratios and dashed lines represented 95% confidence intervals. Model 1: Unadjusted. Model 2: Model 1 + age + gender. Model 3: Model 2 + BMI, ethnicity, residential region, annual household income, physical activity, smoking status, alcohol drinking, chronic disease, meat intake, vegetable intake, fish, dairy products.

Figure 3 demonstrates the dose–response relationships between egg consumption and mortality in analyses using three models. Intuitively, all three models showed an approximately inverted J-shaped dose–response manner. A significant nonlinear relationship was discovered in Model 1 and Model 2, and *p* for nonlinearity was <0.001 for both, while our multi-variate analysis did not show significant violations of the linear assumption (*p* nonlinear = 0.122). The risk of mortality associated with egg intake decreased sharply in the group consuming 0–3/week, with the lowest risk occurring between 3–6/week; afterwards, the risk was not significantly increased in the higher intake group (≥7/week).

Table 2 indicates stratified analyses by participant’s characteristics. Overall, there were no significant interactions between egg intake and covariates (*p* for interaction > 0.1). The reduced risk of death associated with egg consumption may only be observed in a few subgroups. For example, HR estimates of non-smokers and non-drinkers were 0.75 (0.61 to 0.92) and 0.81 (0.68 to 0.96) for the group consuming 3–6/week, respectively. Egg-related mortality risk was similar among participants with BMI <24 kg/m^2^ (0.83 [0.71 to 0.98]).

Sensitivity analyses of egg-mortality associations remained largely robust. After excluding deaths confirmed within the initial first year, individuals aged less than 30 years, or cases diagnosed with chronic disease at baseline, the significant associations of egg intake with mortality did not alter materially (Tables S2–S4). For instance, restricting our analysis to participants without prevalent chronic diseases, HR estimates altered from 0.82 [0.69 to 0.98] to 0.76 [0.62 to 0.93] for ≥7/week (Table S4).

## 4. Discussion

The current investigation is a nearly nationally representative population recruiting 30,835 adult men and women aged 16+ years from 25 provinces and municipalities in mainland China. We observed egg intake was significantly associated with a lower risk of mortality, with an approximately inverted J-shaped dose–response relationship. Our study may provide new evidence to further elucidate the health effects of egg consumption and might influence regional policies on healthy eating habits.

Existing prospective cohort studies on the association of egg consumption with the risk of mortality remain largely heterogeneous worldwide. Our study shows egg consumption reduces the risk of mortality by 17%, which is inconsistent with most studies from the United States [18] and Europe [10,19]. Among other Asian populations, although a Japanese study followed for 14 years reported that participants consuming 1–2 eggs/week had a 22% lower risk of total mortality [20], two other investigations from Japan [21,22] and a large prospective study in Korea [23] showed a significantly increased risk of death from egg intake. Some existing evidence from US cohort studies linked egg intake with an increase of 7–23% in the risk of all-cause mortality [24,25,26,27]. This positive association was also found in a longitudinal analysis involving 20,562 Italian adults (1.50 [1.13–1.99]) [10] and a prospective cohort from the United Kingdom followed for 22.8 years (1.44 [1.14–1.80]) [11]. However, an international prospective study including 177,000+ individuals from 50 countries suggested that moderate egg intake (1/d) was not associated with an increased risk of mortality [28]. Given that egg consumption may vary depending on ethnicities, lifestyle factors and dietary patterns in different regions, the associations with all-cause mortality need to be validated among diverse populations in future prospective studies [29,30].

The longitudinal evidence regarding egg consumption with mortality among Chinese populations is limited. Echoed with our findings, only one nationally representative study has examined the associations of egg intake with all-cause mortality in China showing a protective effect of egg consumption (3–7 eggs/week, 1–2 eggs/day or ≥2 eggs/day) [31]. These comparable results may suggest that moderate-to-high egg intake is worthy of recommendation for the prevention of adverse health outcomes among the Chinese population, which may vary slightly from the latest Chinese dietary guidelines recommending 7 eggs/week [32]. Notably, our study found an approximately J-shape association between egg intake and mortality, with the lowest risk in the moderate-to-high intake group. Another prospective cohort from the China-PAR project found a U-shaped association between egg intake and all-cause mortality, indicating the beneficial effect of moderate egg intake (3–6 eggs/week) and the potential adverse effects in higher or lower egg intake [33]. This inconsistency in findings is probably due to the limited number of participants consuming ≥7 times/week in our study. Regionally, a community-based cohort study conducted in Anhui Province observed a positive relationship between egg consumption and all-cause mortality, and an investigation from the Guangzhou Biobank Cohort Study in China showed null association [34,35]. Based on the results of nationally representative studies, moderate egg intake could benefit public health, but the findings may not be fully applicable in some specific regions. In the future, further research might be needed to formulate suitable dietary guidelines for different regions or specific populations.

To date, increasing evidence has documented underlying biological mechanisms regarding potential beneficial effects of egg consumption on overall health. Egg intake is an important source of omega-3 polyunsaturated fatty acids, which have favorable anti-inflammatory properties and potential therapeutic effects in various chronic inflammatory diseases [36]. Eggs also contain rich carotenoids such as lutein and zeaxanthin, which might have beneficial effects on health throughout the life cycle due to their potential antioxidant properties, as well as its ability to act as a precursor of vitamin A [37]. Egg-derived phospholipids could maintain the balance of lipid metabolism through a compensatory mechanism [38,39], which further alleviates the progress of atherosclerosis [40]. A randomized controlled trial suggested that whole egg consumption further improved atherogenic lipoprotein profile and insulin sensitivity in individuals with metabolic syndrome compared with yolk-free egg substitute [41]. In fact, cholesterol is the main component of egg yolks [42]; some laboratory studies provide evidence that dietary cholesterol may be associated with postprandial inflammation, oxidative stress-related responses and impaired endothelial function [43,44,45]. A cross-over research showed that this phenomenon is more susceptible in high responders [46], an effect that may not have a substantial influence in healthy people [47]. In these circumstances, more research on the physiological mechanisms is warranted to further explore the association between egg consumption and mortality.

There was no significant interaction in the association of egg consumption with the risk of mortality, but our subgroup analysis showed BMI and behavioral factors may be potential effect modifiers. For the middle and highest categories, egg consumption was associated with a lower risk of mortality among participants with BMI <24 kg/m^2^. Likewise, the CKB study, a prospective cohort of approximately 0.5 million participants, observed that the risk appears to decrease by 11–16% in adults with lower or normal BMI [48]. Another 14-year follow-up study showed a higher risk of egg-induced mortality only occurring in BMI ≥21.2 kg/m^2^ [34]. Additionally, we found a lower risk of mortality with moderate egg intake (3–6 times/week) in non-smokers and non-drinkers only. Whereas a population-based cohort study covering 521,120 participants from six US states discovered an 8% increased risk of all-cause death among non-smokers [8]. Due to the inconsistency of the above findings, it is possible that the modification effect by BMI and behavioral factors is a chance finding. The results of subgroup analysis in this study should be interpreted with caution and further validation by other studies is warranted.

The strength of our study is that this is one of the few nationally representative longitudinal cohort studies providing new evidence on the relationship between egg intake and mortality in the Chinese general population. Several limitations should also be noted in this study. First, measurement errors concerning egg intakes assessed through self-reported data were inevitable [7]. Given the lack of detailed information about the actual number of egg intake, our dose–response relationships may not quantitatively capture the real association of egg consumption with mortality well. Second, information about death outcomes in this study were ascertained from their family members at each follow-up visit, which might inevitably induce recall bias to some extent. Third, we failed to explore the association between cause-specific mortality and egg intake due to the data availability. Fourth, though a variety of potential confounding effects on socio-demographic characteristics, lifestyle factors and dietary factors were controlled, residual confounding by different food intake, the cooking methods of eggs and changes in dietary habits during follow-ups may still exist.

## 5. Conclusions

In summary, this nationally representative study provided novel cohort evidence between egg consumption and the risk of mortality in Chinese adults. An approximately inverted J-shaped association between egg intake and mortality was observed with the lowest risk in the moderate-to-high egg intake group. Our findings supported a moderate-to-high egg intake might be recommended as part of healthy dietary habits for the general Chinese adults and could be taken into account in the formulation of public health policies to improve long-term health and longevity.

## Figures and Tables

**Table 1:** Baseline characteristics of included participants (*n* = 30,835) by egg consumption.

		Frequency of Egg Consumption, Times/Week			
Characteristics	Total	0	1–2	3–6	≥7
Population					
Persons, *n*	30,835	7269	8561	9445	5560
All-cause death, *n*	1651	511	465	419	256
Demographic characteristics					
Age, years	45.6 ± 16.3	47 ± 16.5	45.6 ± 15.8	44.5 ± 16.3	45.8 ± 16.6
Male sex, %	48.5	46.3	49.1	49.4	49.3
BMI, kg/m^2^	21.5 ± 5.2	20.7 ± 5.9	21.4 ± 5.4	22 ± 4.5	22.2 ± 4.6
Han ethnicity, %	91.7	85.6	91.0	94.1	96.4
Urban, %	43.7	30.8	40.7	49.8	54.9
Annual household income, %					
Low	28.7	42.1	30.2	22.6	19.1
Medium	43.0	40.7	44.6	43.6	42.2
High	28.4	17.1	25.2	33.8	38.7
Behavioral factors					
Meat intake, %					
0 times/week	18.2	33.7	15.2	12.4	12.3
1 to 3 times/week	45.6	39.3	52.6	45.3	43.2
≥4 times/week	36.2	27.0	32.2	42.3	44.5
Vegetable intake, %					
0 times/week	4.9	13.5	2.5	2.0	2.2
1 to 7 times/week	46.5	44.3	49.0	47.8	43.4
≥8 times/week	48.6	42.2	48.5	50.1	54.4
Fish intake, %					
0 times/week	48.5	72.7	45.5	39.8	36.3
1 times/week	24.7	12.3	30.5	26.7	28.7
≥2 times/week	26.8	15.0	24.0	33.4	35.0
Dairy products, %					
0 times/week	71.6	89.7	76.1	64.3	53.4
1 to 3 times/week	14.6	4.9	16.0	19.1	17.5
≥4 times/week	13.8	5.4	7.8	16.5	29.1
Smoking status, %					
Current	30.4	30.7	32.1	29.5	29.2
Former	6.3	5.0	6.8	6.1	7.5
Never	63.3	64.4	61.1	64.5	63.4
Alcohol consumption, %					
Current	16.1	14.6	15.6	16.3	18.3
Former	4.6	4.6	4.6	4.1	5.7
Never	79.3	80.8	79.8	79.7	76.0
Physical activity, %					
0 min/week	73.3	80.6	76.8	70.0	63.7
1 to < 60 min/week	23.2	17.3	20.3	25.8	30.9
≥60 min/week	3.5	2.1	2.9	4.2	5.4
Health status					
Chronic diseases, %	14.7	16.3	15.6	13.0	14.4
Cardiovascular disease	3	2.9	2.9	3.0	3.3
Respiratory diseases	1.6	1.7	1.8	1.5	1.7
Diabetes mellitus	0.7	0.6	0.6	0.7	0.8
Cancer	0.2	0.2	0.3	0.2	0.1

Data are presented using mean ± SD for continuous variables and percentages for categorical variables. BMI = body mass index.

**Table 2:** Subgroup analysis for association of egg consumption with all-cause mortality.

			Hazard Ratio ᵃ [95%CI]			*p* for Trend	*p* for Interaction
Subgroup	Participants	Death	1–2 Times/Week	3–6 Times/Week	≥7 Times/Week		
Gender							0.152
Male	14,970	1007	1.04 [0.87–1.25]	0.85 [0.71–1.03]	0.77 [0.62–0.97] *	0.005	
Female	15,865	644	0.82 [0.64–1.04]	0.81 [0.64–1.03]	0.93 [0.70–1.23]	0.404	
Age, years							0.403
16–49	18,382	233	1.00 [0.69–1.45]	0.89 [0.60–1.32]	0.67 [0.40–1.12]	0.127	
≥50	12,453	1418	0.95 [0.82–1.12]	0.84 [0.71–0.98] *	0.85 [0.71–1.03]	0.026	
BMI, kg/m^2^							0.917
<24	21,744	1207	0.94 [0.80–1.10]	0.83 [0.71–0.98] *	0.80 [0.66–0.97] *	0.007	
≥24	9091	444	1.00 [0.71–1.41]	0.87 [0.62–1.24]	0.91 [0.63–1.34]	0.462	
Residential region							0.198
Urban	13,479	614	0.81 [0.63–1.05]	0.86 [0.68–1.10]	0.78 [0.60–1.02]	0.127	
Rural	17,356	1037	1.04 [0.87–1.24]	0.82 [0.68–0.99] *	0.86 [0.68–1.08]	0.033	
Smoking status							0.341
No	19,523	824	0.83 [0.67–1.02]	0.75 [0.61–0.92] **	0.81 [0.63–1.03]	0.025	
Yes	11,312	827	1.09 [0.89–1.34]	0.94 [0.76–1.16]	0.84 [0.66–1.08]	0.082	
Alcohol consumption							0.112
No	24,449	1193	0.86 [0.72–1.01]	0.81 [0.68–0.96] *	0.82 [0.67–1.01]	0.026	
Yes	6386	458	1.28 [0.96–1.70]	0.90 [0.66–1.22]	0.89 [0.64–1.24]	0.129	
Physical activity							0.723
No	22,587	1231	0.95 [0.80–1.12]	0.84 [0.71–1.00] *	0.87 [0.71–1.07]	0.057	
Yes	8248	420	0.95 [0.70–1.31]	0.82 [0.60–1.13]	0.70 [0.50–0.99] *	0.021	

ᵃ We adjusted gender, age, BMI, ethnicity, residential region, annual household income, physical activity, smoking status, alcohol drinking, chronic disease, meat intake, vegetable intake, fish, dairy products. HR: hazard ratio; 95% CI: 95% confidence interval. Notes: * *p* < 0.05; ** *p* < 0.01.

## Data Availability

The data presented in this study are accessible on the Open Research Data Platform and the China Family Panel Study (CFPS) official Web site of Peking University (https://www.isss.pku.edu.cn/cfps/, accessed on 13 August 2021).

## Supplementary Materials

The following supporting information can be downloaded at: https://www.mdpi.com/article/10.3390/ijerph192214777/s1, Figure S1: Inclusion and exclusion flow chart of study sample; Table S1: Association between egg consumption and mortality; Table S2: Association between egg consumption and mortality in the CFPS after excluding cases of death that occurred within the first year of follow-up; Table S3: Association between egg consumption and mortality in the CFPS after excluding cases aged less than 30 years; Table S4: Association between egg consumption and mortality in the CFPS after excluding cases diagnosed with chronic disease at baseline.
