# Supplementary material for: Frequency of Egg Intake Associated with Mortality in Chinese Adults: An 8-Year Nationwide Cohort Study
Source: Int J Environ Res Public Health. 2022 Nov 10;19(22):14777. doi: 10.3390/ijerph192214777 (PMC9690384; doi:10.3390/ijerph192214777)
Supplement: Supplementary file 1 [file ijerph-19-14777-s001.zip › ijerph-1904837-supplementary.pdf]

Supplementary Material

Frequency of Egg Intake Associated with Mortality in Chinese Adults: An 8-Year Nationwide Cohort Study

Ke Wang [1,†], Qianqian Xiang [2,†], Lan Hu [1,3], Lu Wang [4,*], Yunquan Zhang [5,6,*]

[1] Department of Nursing, Medical College, Wuhan University of Science and Technology, Wuhan 430065, China

[2] Hubei Provincial Center for Disease Control and Prevention, Wuhan, 430079, China

[3] Department of Head and Neck Surgery, Hubei Cancer Hospital, Tongji Medical College, Huazhong University of Science and Technology, Wuhan 430079, Hubei, China

[4] School of Basic Medicine, Hubei University of Arts and Sciences, Xiang yang, Hubei 441053, China

[5] Department of Epidemiology and Biostatistics, School of Public Health, Wuhan University of Science and Technology, Wuhan 430065, China

[6] Hubei Province Key Laboratory of Occupational Hazard Identification and Control, Wuhan University of Science and Technology, Wuhan 430065, China

*Corresponding author: Y. Zhang, Department of Epidemiology and Biostatistics, School of Public Health, Wuhan University of Science and Technology, Wuhan 430065, China. E-mail: YunquanZhang@wust.edu.cn

*Corresponding author: L. Wang, School of Basic Medicine, Hubei University of Arts and Sciences, Xiang yang, Hubei 441053, China. E-mail: 11706@hbuas.edu.cn

[†] These authors contributed equally to this work.

Table of contents

Figure S1. Inclusion and exclusion flow chart of study sample.
Table S1. Association between egg consumption and mortality.
Table S2. Association between egg consumption and mortality in the CFPS after excluding cases of death that occurred within the first year of follow-up.
Table S3. Association between egg consumption and mortality in the CFPS after excluding cases aged less than 30 years.
Table S4. Association between egg consumption and mortality in the CFPS after excluding cases diagnosed with chronic disease at baseline.

Figures

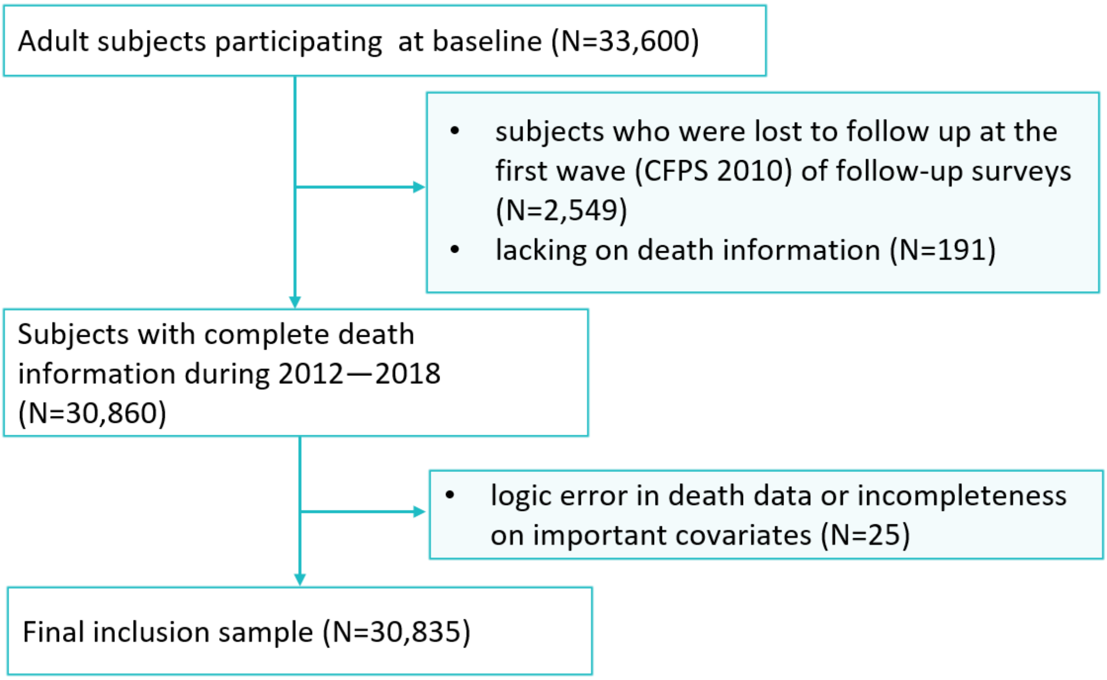

Figure S1. Inclusion and exclusion flow chart of study sample.

Tables

Table S1. Association between egg consumption and mortality.

	Hazard ratio [95% CI]				
**Model**	**0 times/week**	**1–2 times/week**	**3–6 times/week**	**≥7 times/week**	**P for trend**
Model 1	1(Ref)	0.77 [0.68 to 0.88] ***	0.64 [0.56 to 0.73] ***	0.66 [0.57 to 0.77] ***	<0.001
Model 2	1(Ref)	0.85 [0.74 to 0.96] **	0.72 [0.63 to 0.82] ***	0.69 [0.59 to 0.81] ***	<0.001
Model 3	1(Ref)	0.95 [0.82 to 1.10]	0.84 [0.72 to 0.97] *	0.82 [0.69 to 0.98] *	0.007

Model 1: Unadjusted.

Model 2: Model 1 + age +gender.

Model 3: Model 2 + BMI, ethnicity, residential region, annual house-hold income, physical activity, smoking status, alcohol drinking, chronic disease, meat intake, vegetable intake, fish, dairy products.

Notes: *p < 0.05; **p < 0.01; ***p < 0.001.

Table S2. Association between egg consumption and mortality in the CFPS after excluding cases of death that occurred within the first year of follow-up.

	Hazard ratio [95% CI]				
**Model**	**0 times/week**	**1–2 times/week**	**3–6 times/week**	**≥7 times/week**	**P for trend**
Model 1	1(Ref)	0.77 [0.68 to 0.88] ***	0.64 [0.56 to 0.73] ***	0.66 [0.57 to 0.77] ***	<0.001
Model 2	1(Ref)	0.85 [0.74 to 0.96] **	0.72 [0.63 to 0.82] ***	0.69 [0.59 to 0.81] ***	<0.001
Model 3	1(Ref)	0.96 [0.83 to 1.11]	0.84 [0.72 to 0.97] *	0.83 [0.70 to 0.99] *	0.007

Model 1: Unadjusted.

Model 2: Model 1 + age +gender.

Model 3: Model 2 + BMI, ethnicity, residential region, annual house-hold income, physical activity, smoking status, alcohol drinking, chronic disease, meat intake, vegetable intake, fish, dairy products.

Notes: *p < 0.05; **p < 0.01; ***p < 0.001.

Table S3. Association between egg consumption and mortality in the CFPS after excluding cases aged less than 30 years.

	Hazard ratio [95% CI]				
**Model**	**0 times/week**	**1–2 times/week**	**3–6 times/week**	**≥7 times/week**	**P for trend**
Model 1	1(Ref)	0.78 [0.69 to 0.89] ***	0.67 [0.58 to 0.76] ***	0.69 [0.59 to 0.80] ***	<0.001
Model 2	1(Ref)	0.86 [0.77 to 0.97] *	0.82 [0.73 to 0.92] **	0.89 [0.78 to 1.01]	<0.001
Model 3	1(Ref)	0.83 [0.73 to 0.94] **	0.79 [0.69 to 0.89] ***	0.82 [0.71 to 0.95] **	0.005

Model 1: Unadjusted.

Model 2: Model 1 + age +gender.

Model 3: Model 2 + BMI, ethnicity, residential region, annual house-hold income, physical activity, smoking status, alcohol drinking, chronic disease, meat intake, vegetable intake, fish, dairy products.

Notes: *p < 0.05; **p < 0.01; ***p < 0.001.

Table S4. Association between egg consumption and mortality in the CFPS after excluding cases diagnosed with chronic disease at baseline.

	Hazard ratio [95% CI]				
**Model**	**0 times/week**	**1–2 times/week**	**3–6 times/week**	**≥7 times/week**	**P for trend**
Model 1	1(Ref)	0.75 [0.65 to 0.87] ***	0.62 [0.53 to 0.72] ***	0.58 [0.48 to 0.69] ***	<0.001
Model 2	1(Ref)	0.84 [0.72 to 0.97] *	0.71 [0.61 to 0.83] ***	0.62 [0.52 to 0.75] ***	<0.001
Model 3	1(Ref)	0.95 [0.81 to 1.12]	0.84 [0.71 to 0.99] *	0.76 [0.62 to 0.93] **	0.003

Model 1: Unadjusted.

Model 2: Model 1 + age +gender.

Model 3: Model 2 + BMI, ethnicity, residential region, annual house-hold income, physical activity, smoking status, alcohol drinking, chronic disease, meat intake, vegetable intake, fish, dairy products.

Notes: *p < 0.05; **p < 0.01; ***p < 0.001.
